# Phylogrouping and characterization of *Escherichia coli* isolated from colonic biopsies and fecal samples of patients with flare of inflammatory bowel disease in Iran

**DOI:** 10.3389/fmed.2022.985300

**Published:** 2022-08-29

**Authors:** Banafsheh Nadalian, Bahareh Nadalian, Hamidreza Houri, Shabnam Shahrokh, Mohammad Abdehagh, Abbas Yadegar, Gholamhossein Ebrahimipour

**Affiliations:** ^1^Department of Microbiology and Microbial Biotechnology, Faculty of Life Sciences and Biotechnology, Shahid Beheshti University, Tehran, Iran; ^2^Foodborne and Waterborne Diseases Research Center, Research Institute for Gastroenterology and Liver Diseases, Shahid Beheshti University of Medical Sciences, Tehran, Iran; ^3^Gastroenterology and Liver Diseases Research Center, Research Institute for Gastroenterology and Liver Diseases, Shahid Beheshti University of Medical Sciences, Tehran, Iran; ^4^Basic and Molecular Epidemiology of Gastrointestinal Disorders Research Center, Research Institute for Gastroenterology and Liver Diseases, Shahid Beheshti University of Medical Sciences, Tehran, Iran

**Keywords:** *Escherichia coli*, inflammatory bowel disease, antimicrobial resistance, phylogrouping, pathotypes, multidrug-resistant phenotype

## Abstract

**Background:**

Although the etiopathogenesis of inflammatory bowel disease (IBD) is still poorly understood, *Escherichia coli* has been described as a potential causative microorganism in IBD pathogenesis and also disease progression, offering a potential therapeutic target for disease management. Therefore, we conducted this study to investigate the pathotypes, phylogenetic groups, and antimicrobial resistance of *E. coli* isolates from patients with IBD in Iran.

**Methods:**

Fecal and biopsy colonic samples were collected from IBD patients experiencing flare-up episodes referred to Taleghani hospital in Tehran, Iran, between August 2020 and January 2021. Identification of *E. coli* strains was performed based on biochemical and molecular methods. Antibiotic susceptibility testing was performed as recommended by the Clinical and Laboratory Standards Institute. Phylogrouping and pathotyping of each isolate were carried out using polymerase chain reaction (PCR) and multilocus sequence typing (MLST) assays.

**Results:**

A total of 132 non-duplicate *E. coli* strains were isolated from 113 IBD patients, including 96 ulcerative colitis (UC), and 17 Crohn’s disease (CD) patients. In our study, 55% of CD-related *E. coli* and 70.5% of UC-related isolates were non-susceptible to at least three or more unique antimicrobial classes, and were considered as multidrug-resistant (MDR) strains. *E. coli* strains exhibited a high level of resistance to cefazolin, ampicillin, tetracycline, ceftazidime, ciprofloxacin, and cefotaxime. Enterotoxigenic *E. coli* (ETEC) and diffusely adherent *E. coli* (DAEC) were the most prevalent pathotypes, and groups B2 and D were the predominant phylogroups.

**Conclusion:**

In the present study, we found that *E. coli* strains that colonize the gut of Iranian patients with IBD most frequently belonged to phylogenetic groups B2 and D. We also conclude that *E. coli* isolates from IBD patients have been revealed to be resistant to commonly used antibiotics, in which most of them harbored strains that would be categorized as MDR.

## Background

Inflammatory bowel disease (IBD), including Crohn’s disease (CD) and ulcerative colitis (UC), are chronic, idiopathic inflammatory disorders characterized by relapsing and remitting episodes of intestinal inflammation (1). CD and UC are distinguished by the location, clinical manifestations, and hypothesized pathogenic mechanisms. The clinical features of IBD patients depend on the site and extent of mucosal inflammation that may include diarrhea, rectal bleeding, abdominal pain, malnutrition, and weight loss (2). The etiopathogenesis of IBD is still poorly understood; however, genetic susceptibility, environmental factors, altered intestinal microbiota, and immune-mediated intestinal injury are thought to be seriously involved in this complex disorder (3).

Altered microbial communities, termed dysbiosis, are associated with changes in microbial abundance, composition and a reduction in the overall biodiversity. Previous studies have suggested a decrease in intestinal species richness, more specifically a significant drop in Firmicutes, and an increase in the prevalence of Proteobacteria phylum, particularly Enterobacteriaceae, in IBD patients (4, 5). Recent researches have also identified *Escherichia coli* as a potential causative microorganism in IBD pathogenesis and also disease progression (6–8).

Based on the genetic and clinical criteria, *E*. *coli* strains can be classified into three major groups, including commensal strains, intestinal pathogenic or diarrheagenic *E. coli* (DEC), and extraintestinal pathogenic *E. coli* (ExPEC) (9). There are six classic pathotypes among the DECs according to the virulence factors they possess or pathological effects they cause, including enteropathogenic *E. coli* (EPEC), Shiga toxin-producing *E. coli* (STEC), enterotoxigenic *E. coli* (ETEC), enteroaggregative *E. coli* (EAEC), enteroinvasive *E. coli* (EIEC) and diffusely adherent *E. coli* (DAEC) (10).

It has been previously described that IBD-associated *E. coli* isolates commonly carry ExPEC-associated genetic markers (11, 12). On the other hand, several lines of evidence indicated that some commensal *E. coli* strains in the gut, which are better described as pathobionts, are associated with immune-mediated disorders like IBD. Importantly, the pathogenicity of these strains are depending on environmental and host genetic factors to cause disease (13, 14). Recently, several additional studies have suggested the involvement of adherent-invasive *E. coli* (AIEC) in the pathogenesis of CD, with demonstrations that these heterogeneous strains can invade human intestinal epithelial cells as well as survive in macrophages, resulting in tissue damage and inflammation (15–19). Although DEC strains have not been comprehensively recognized to implicate in the pathogenesis of IBD, some authors have described that they may promote intestinal inflammation (14). They have also shown that DAEC strains possessing adherence factors reside in the large intestine and can attach to the rectal mucosa, irrespective of the presence of colitis in UC patients (14).

Among typing methods applied for characterization of *E. coli* strains, such as pulsed-field gel electrophoresis (PFGE), enterobacterial repetitive intergenic consensus sequence (ERIC)-PCR, randomly amplified polymorphic DNA (RAPD), ribotyping, multiplex phylogrouping PCR has been widely employed because of its simplicity and rapidity (20–22). Phylogrouping technique is also useful for genetic structure analysis of *E. coli* populations, classification of extraintestinal pathogenic and commensal strains, and also host-source relationships (23). Additionally, it has been reported that different phylogroups vary in the presence of virulence factors, ecological niches and their antibiotic-resistance profiles (24). *E*. *coli* isolates from IBD patients frequently belong to certain phylogroups, particularly phylogroups B2 and D, compared to healthy controls (12).

Since the 1970s, various investigators have described increased numbers of *E. coli* isolates with certain virulence characteristics from IBD patients compared to those from healthy controls, particularly when focusing on CD patients during disease relapses (8, 25–27). For instance, the adhesion index of active CD-associated *E. coli* isolates was significantly higher than those isolated from healthy subjects (8). The adhesion capabilities of *E. coli* allow the bacterium to colonize the intestinal mucosa and induce intestinal inflammation (18). In addition, it was found that hemolysin- and necrotoxin-producing *E. coli* were associated with relapse of UC, however, the authors suggested that the inflammation during a relapse of colitis tends to favor the presence of these organisms, rather than that these organisms cause the relapse (28). Moreover, most IBD-associated *E. coli* isolates showed higher invasion properties than those from healthy subjects, in which the invasive properties are associated with induction of bowel inflammation (18, 29).

The frequent recovery of *E. coli* strains possessing certain virulence markers from patients with CD and UC has increased interest in these strains over the last two decades (6, 19, 29). However, there are only very limited data reported on the association between *E. coli* and IBD pathogenesis in Iran (30, 31). With mounting evidence confirming the impact of dysbiotic microbiome in IBD, some antibiotics are exploited for treating bacterial overgrowth, as well as for the treatment of IBD flare-up and septic complications of the disease, such as abscesses and post-operative wound infections. In this regard, different antibiotics are used as empirical antibiotic therapy for IBD patients, most often ciprofloxacin and metronidazole, each alone or in combination (32). The aim of this study principally was to investigate the pathotypes, phylogenetic groups, and antimicrobial resistance of *E. coli* isolates from patients with IBD in Iran.

## Materials and methods

### Patients, biopsies, and fecal samples

In this cross-sectional study, 156 patients with confirmed diagnosis of IBD who referred to Taleghani hospital in Tehran, Iran were enrolled in this study between August 2020 and January 2021. A combination of clinical, radiological, endoscopic, and pathological criteria was considered for a reliable diagnosis of IBD (33). Biopsy specimens were obtained from 56 IBD patients during colonoscopy, and 100 fecal samples were collected from the rest of the patients. All the biopsies were taken from inflamed tissue from the ileum and/or colon. Demographic and disease variables such as; age, gender, disease duration, medication, and clinical features were recorded for all patients through a questionnaire. The flare-up of CD and UC patients was diagnosed according to Crohn’s disease activity index (CDAI) and Powell-Tuck index, respectively (34). This study was given ethical approval by the Institutional Ethical Review Committee of Research Institute for Gastroenterology and Liver Diseases (RIGLD) at Shahid Beheshti University of Medical Sciences (Project No. IR.SBMU.RIGLD.REC.1399.052). Informed consent was obtained from all subjects and/or their legal guardians prior to sample collection.

### Bacterial isolation and identification

Biopsy samples were taken with sterile forceps, placed in brain heart infusion (BHI) broth solution, and then immediately delivered to the microbiology laboratory of Foodborne and Waterborne Diseases Research Center, Research Institute for Gastroenterology and Liver Diseases (35). The fresh biopsy samples were homogenized in BHI broth, then the tissue homogenates were plated on MacConkey agar and incubated for 24 h at 37°C in aerobic conditions. Subsequently, single pink (lactose fermenting) colonies on MacConkey agar were picked for further confirmation (36).

The freshly collected stool sample was immediately transported to the microbiology laboratory for the microbial examination within 1 h of collection. Briefly, fecal sample suspension was prepared in phosphate buffer solution (PBS) and plated onto MacConkey agar followed by 24 h incubation at 37°C aerobically (37). The identification of *E. coli* strains was done based on morphological and biochemical tests (38). Additionally, molecular confirmation was performed by PCR on *E. coli* 16S rRNA gene using specific primers (ECB75F: 5′-GGAAGAAGCTTGCTTCTTTGCTG-3′, ECR620R: 5′-GAGCCCGGGGATTTCACAT-3′) as previously described (39).

### Antimicrobial susceptibility testing

Antibiotic susceptibility testing was performed using the Kirby–Bauer disk diffusion method as recommended by the Clinical and Laboratory Standards Institute (CLSI) guidelines (40). Commercially available antibiotic disks (Mast Co., United Kingdom) used in this study included ampicillin (10 μg), piperacillin/tazobactam (100/10 μg), cefazolin (30 μg), cefoxitin (30 μg), cefotaxime (30 μg), ceftazidime (30 μg), cefepime (30 μg), imipenem (10 μg), aztreonam (30 μg), ciprofloxacin (5 μg), ofloxacin (5 μg), gentamicin (10 μg), amikacin (30 μg), tetracycline (30 μg). In addition, susceptibility to colistin was performed using minimum inhibitory concentration (MIC). Since there are no CLSI breakpoints for Enterobacteriaceae for MIC testing of colistin, its MICs were interpreted based on the European Committee on Antimicrobial Susceptibility Testing (EUCAST) guidelines as follows: ≤2 mg/L, susceptible; >2 mg/L, resistant (41). Multidrug-resistant (MDR) phenotype was defined as non-susceptibility to at least one agent in three or more antimicrobial classes (42).

### DNA extraction

Total genomic DNA of *E. coli* isolates was extracted from the fresh culture of the bacterium using the QIAamp DNA Mini Kit (QIAGEN, Hilden, Germany) according to the manufacturer’s instructions. The DNA concentration was measured by NanoDrop ND-1000 spectrophotometer (Thermo Scientific, Waltham, MA, United States) and the integrity of DNA was evaluated using electrophoresis on 0.8% (w/v) agarose gels. DNA samples were kept at −20°C until used for PCR assays.

### Virulence detection and pathotyping of *E. coli* isolates

Molecular characterization of *E. coli* pathotypes was carried out based on virulence gene detection of all 6 categories of intestinal pathogenic *E. coli* by PCR method as described previously (43–45). Genes used to screen for identification of different pathotypes include *lt* and *stII* for ETEC, *eae* for atypical (aEPEC), *eae* and *bfp* for typical EPEC, *aggR* and *pvcD* for EAEC, *stx*_1_ and *stx*_2_ for EHEC, *virF* and *ipaH* for EIEC, and *daaD* for DAEC. The reaction mixture contained 12.5 μl of Taq DNA Polymerase Master Mix (Ampliqon, Denmark), 1 μl (10 pM/μl) of each primer, 8.5 μl of distilled water, and 2 μl (100 ng) of DNA template in a final volume of 25 μl. PCR reactions were performed under the following conditions: 96°C for 4 min, 94°C for 20 s, 55°C for 20 s, and 72°C for 10 s for 30 cycles, with a final extension at 72°C for 7 min. PCR products were visualized following electrophoresis through 1.5% agarose gel (Gibco Life Technologies, Paisley, United Kingdom) stained with ethidium bromide.

### *E. coli* phylogrouping

The phylogroup of each isolate was determined based on *E. coli* phylogrouping method described by Clermont et al. (46). Briefly, this method assigns strains to phylogroups A, B1, B2, C, D, E, F that belong to *E. coli sensu stricto*, whereas the eighth is the *Escherichia* cryptic clade I. This technique has been designed based on extended quadruplex PCR and multilocus sequence typing (MLST) scheme (47, 48). All PCR reactions were carried out in a 20 μl final volume containing 2 μl of 10X buffer, 2 μM of dNTPs, 2 U of Taq polymerase (Ampliqon, Denmark), 2 μl (100 ng) of DNA template and the appropriate primers. The amounts of primer used are 20 pmol, except for AceK.f (40 pmol), ArpA1.r (40 pmol), trpBA.f (12 pmol) and trpBA.r (12 pmol). PCR reactions were performed under the following conditions: denaturation 4 min at 94°C, 30 cycles of 5 s at 94°C and 20 s at 57°C (group E) or 59°C (quadruplex and group C), and a final extension step of 5 min at 72°C. The primers used for the allele-specific phylogroups E and C PCRs were ArpAgpE.f and ArpAgpE.r and trpAgpC.f and trpAgpC.r, respectively. In E- and C-specific PCR reactions, the primers trpBA.f and trpBA.r are added to provide an internal control.

### Statistical analysis

Data analysis was performed using IBM SPSS Statistics 20.0 (IBM Corp., Armonk, NY, United States) and GraphPad Prism software version 8 (GraphPad Software, Inc., CA, United States). Categorical variables among groups were compared using the Chi-square test. Results were presented as the average ± standard error of the mean (SEM) of at least three experiments unless otherwise stated. Differences were considered statistically significant when *P < 0.05.

## Results

### Baseline demographics and clinical characteristics

One hundred and fifty-six IBD patients experiencing flare-ups consisting of 129 (82.7%) UC and 27 (17.3%) CD were enrolled in this study. The median ages of UC and CD patients were 36.08 ± 14.37 years and 39.32 ± 17.22 years, respectively. Proctitis was the most common extent of disease among UC patients (65, 50.4%), followed by left-sided colitis (21, 16.3%), backwash ileitis (15, 11.6%), and pancolitis (11, 8.5%). The ileocolonic region was the most predominantly affected area among CD patients, followed by the right-sided colitis. More detailed demographic and clinical characteristics of the patients are summarized in Table 1. According to the Chi-square test there were no significant differences (*P* > 0.05) between CD and UC patients regarding to the demographic and clinical characteristics.

**TABLE 1:** Demographic data and clinical characteristics of the inflammatory bowel disease (IBD) patients.

Characteristics	UC (*n* = 129)	CD (*n* = 27)	Total (*n* = 156)
**Age range** 1–18 19–30 31–50 51–60 61–70 71–80	7 (5.4) 42 (32.6) 53 (41.1) 13 (10.1) 10 (7.7) 4 (3.1)	1 (3.7) 5 (18.5) 14 (51.9) 4 (14.8) 2 (7.4) 1 (3.7)	8 (5.1) 47 (30.1) 67 (42.9) 17 (10.9) 12 (7.7) 5 (3.2)
**Sex** Female Male	71 (55) 58 (45)	15 (55.6) 12 (44.4)	86 (55.1) 70 (44.9)
**Antibiotic use** Yes No	68 (52.7) 61 (47.3)	11 (40.7) 16 (59.3)	79 (50.6) 77 (49.4)
**IBD drugs** Mesalazine Sulfasalazine Azathioprine CinnoRA Prednisolone Infliximab	67 (52) 25 (19.4) 37 (28.7) 11 (8.5) 24 (18.6) 10 (7.8)	8 (29.6) 6 (22.2) 7 (25.9) 1 (3.7) 2 (7.4) 0 (0)	75 (48.1) 31 (19.9) 44 (28.2) 12 (7.7) 26 (16.7) 10 (6.4)
**Extent of disease** Proctitis Left-sided colitis Backwash ileitis Pancolitis Ileocolitis Right-sided colitis	65 (50.4) 21 (16.3) 15 (11.6) 11 (8.5) - -	- - - - 23 (85.2) 4 (14.8)	75 (58.1) 24 (18.6) 17 (13.2) 13 (10.1) 23 (85.2) 4 (14.8)
**Smoking** Yes No	15 (11.6) 114 (88.4)	5 (18.5) 22 (81.5)	20 (12.8) 136 (87.2)

IBD, inflammatory bowel disease, UC, ulcerative colitis, CD, Crohn’s disease.

### *E. coli* isolates and antibiotic resistance patterns

**FIGURE 1:**
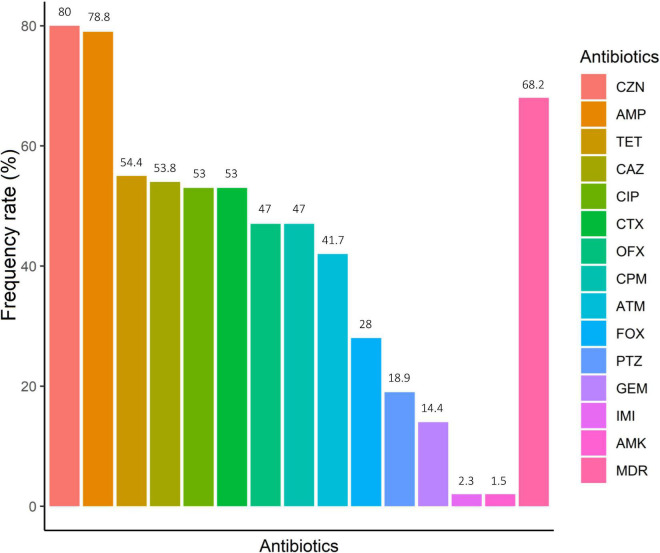
Antimicrobial resistance of IBD-associated *E. coli*. Amikacin (AMK), ampicillin (AMP), cefotaxime (CTX), piperacillin/tazobactam (PTZ), cefazolin (CZN), cefoxitin (FOX), ceftazidime (CAZ), cefepime (CPM), imipenem (IMI), aztreonam (ATM), ciprofloxacin (CIP), ofloxacin (OFX), gentamicin (GEM) and tetracycline (TET). *MDR, multidrug-resistant.

A total of 132 non-duplicate *E. coli* strains were isolated from 113 IBD patients, including 96 UC and 17 CD patients. Of these, 47 strains were recovered from 39 biopsy samples and 85 from 74 stool samples. From 12 patients with UC and 3 with CD at least two different *E. coli* strains from each patient were recovered based on pathotyping, phylogenetic typing, and/or antibiotic resistance patterns. Table 2 shows the antimicrobial resistance patterns according to disease type and sample type. *E. coli* isolates were highly resistant to cefazolin (80.3%, *n* = 106), ampicillin (78.8%, *n* = 104), and tetracycline (54.5%, *n* = 72) (Figure 1). All isolates were susceptible to colistin. The concentrations that inhibited 50% (MIC50) and 90% (MIC90) of colistin for *E. coli* isolates were 0.125 and 0.25 μg/mL, respectively. In our study, 55% of CD- and 70.5% of UC-associated *E. coli* isolates were non-susceptible (intermediate or resistant) to at least three or more unique antimicrobial classes and, thus were considered as MDR strains. Resistance to ciprofloxacin, an antibiotic commonly selected for use in IBD patients, was present in 50% of CD- associated and 72% of UC- associated *E. coli* strains. Chi-square test revealed that there was no significant difference (*P* > 0.05) between CD- and UC- *E. coli* isolates with regarding to the antimicrobial patterns.

**TABLE 2:** Antimicrobial resistance profile (including resistant and intermediate) of the IBD-associated *E. coli* isolates according to the type of the disease.

Antibiotics	UC (*n* = 112)		CD (*n* = 20)	
	Stool isolates (*n* = 77)	Biopsy isolates (*n* = 35)	Stool isolates (*n* = 8)	Biopsy isolates (*n* = 12)
Piperacilline/Tazobactam	21 (27.3)	12 (34.3)	2 (25)	7 (58.3)
Cefazolin	75 (97.4)	34 (97.1)	8 (100)	11 (91.6)
Ciprofloxacin	54 (70.1)	27 (77.1)	1 (12.5)	9 (75)
Gentamicin	10 (13)	6 (17.1)	1 (12.5)	3 (25)
Cefoxitin	35 (45.5)	17 (48.6)	2 (25)	4 (33.3)
Cefepime	44 (57.1)	28 (80)	3 (37.5)	12 (100)
Cefotaxime	51 (66.2)	23 (65.7)	4 (50)	9 (75)
Tetracycline	46 (59.7)	23 (65.7)	3 (37.5)	6 (50)
Amikacin	1 (1.3)	6 (17.1)	0 (0)	0 (0)
Aztreonam	36 (46.8)	17 (48.6)	1 (12.5)	6 (50)
Ofloxacin	42 (54.5)	17 (48.6)	1 (12.5)	5 (41.7)
Ceftazidime	43 (55.8)	21 (60)	3 (37.5)	8 (66.6)
Imipenem	4 (5.2)	2 (5.7)	0 (0)	0 (0)
Ampicillin	64 (83.1)	30 (85.7)	5 (62.5)	11 (91.6)

IBD, inflammatory bowel disease, UC, ulcerative colitis, CD, Crohn’s disease.

### Pathotyping, phylogrouping, and multilocus sequence typing

*E. coli* isolates were studied concerning virulence factors and pathotypes, in which *lt*, *daaD*, *pvcD*, *aggR*, *eae* genes were detected in 21 (15.9%), 11 (8.3%), 3 (2.3%), 3 (2.3%), and 1 (0.8%) strains, respectively. No STEC strain was detected among *E. coli* isolates examined, while, one EPEC, 21 ETEC, 11 DAEC, and 3 EAEC strains were isolated. All EAEC strains were cultured from patients with UC, whereas DAEC was recovered from CD patients with a higher frequency than UC subjects, however these differences did not reach statistical significance.

**FIGURE 2:**
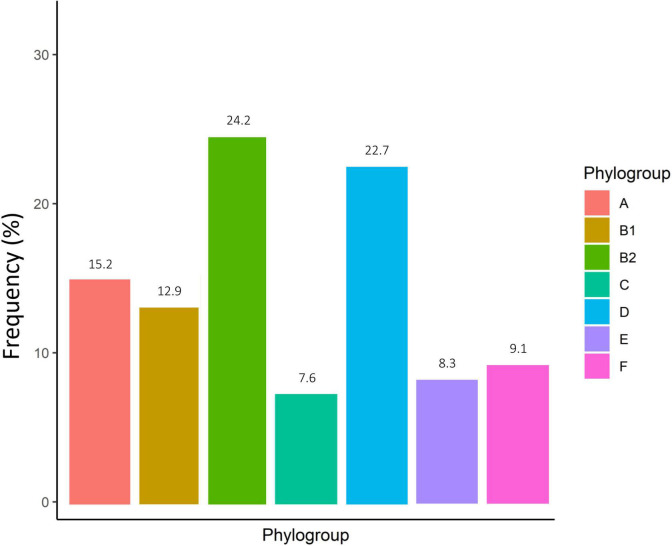
Frequency of phylogenetic groups of *E. coli* isolates in Iranian IBD patients.

**FIGURE 3:**
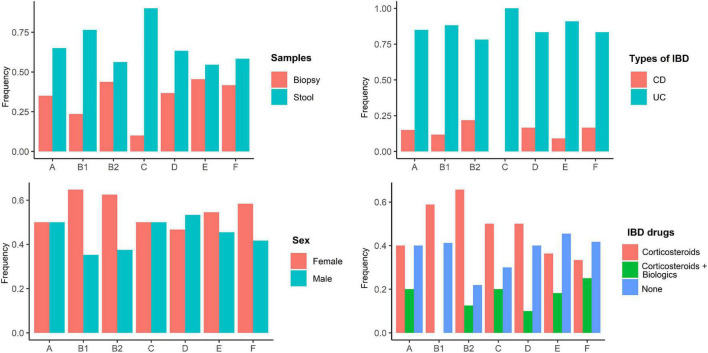
Frequency of phylogenetic groups according to the gender of patients, type of samples, type of disease, and the used biologics and anti-inflammatory drugs.

**FIGURE 4:**
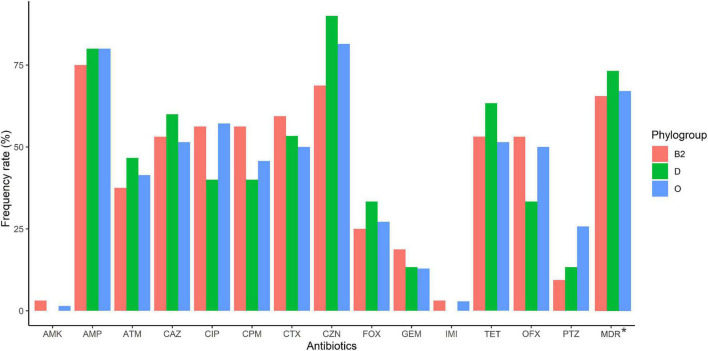
Antimicrobial resistance of IBD-associated *E. coli* isolates according to phylogroup (B2, D, and other than B2 and D [O]). Amikacin (AMK), ampicillin (AMP), cefotaxime (CTX), piperacillin/tazobactam (PTZ), cefazolin (CZN), cefoxitin (FOX), ceftazidime (CAZ), cefepime (CPM), imipenem (IMI), aztreonam (ATM), ciprofloxacin (CIP), ofloxacin (OFX), gentamicin (GEM) and tetracycline (TET).

According to the genomic similarity analysis using phylogrouping, phylogroup B2 (24.2%: 20.5% in UC and 30% in CD) and phylogroup D (22.7%: 17.8% in UC and 25% in CD) were the most prevalent phylogroups among the IBD patients. The percentage of other phylogroups were determined 15.2%, 12.9%, 9.1%, 8.3%, 7.6% for A, B1, F, E, and C, respectively. In addition, 6 isolates could not be assigned to none of the phylogroups using quadruplex PCR assay, which then were characterized using MLST. We found six ST types including ST1049, ST10, ST2817, ST12, ST1279, and ST74 allocated to phylogroups B2, A, A, B2, B1, and B2 respectively. Figure 2 and Supplementary Table 1 represent the distribution of *E. coli* strains according to the phylogrouping and MLST analysis. Analysis of the phylogroup distribution of *E. coli* strains and demographics of IBD patients showed no significant correlations. Figure 3 represents the frequency of phylogenetic groups according to the gender of patients, type of samples, type of disease, and the used biologics and anti-inflammatory drugs. Figure 4 depicts resistance to commonly used antibiotics according to the phylogenetic groups.

## Discussion

Several independent investigations have suggested a considerable increase in a specific population of *E. coli* belonging to phylogroup B2 among patients with IBD (14). However, some animal studies have described overgrowth of *E. coli* after DSS-induced colitis, which suggest that *E. coli* enrichment in the gut can be as a consequence of inflammation (19). Any answer concerning the role of *E. coli* in the pathogenesis of IBD needs isolation and characterization of this bacterium for further in-depth analysis of host-microbe interactions. Moreover, much of the research on these *E. coli* strains, as well as their potential impact in IBD, has been performed in Western countries (Europe, North America, and Australia), however there is insufficient data regarding the characterization of *E. coli* isolated from CD and UC patients in the Middle East. It is currently not well comprehended whether the overgrowth of such putative pathogens described among IBD populations is more widespread in the patients in regions with increasing IBD incidence, especially in the Middle East countries and more specifically in Iran. In the current study, we performed phylogenetic analysis and our findings demonstrated that most *E. coli* strains were classified into phylogenetic groups B2 (24.2%) and D (22.7%), which are reported to be genetically similar to ExPEC strains (49). The strains belonged to phylogruops B2 and D harbor *chuA* gene, which is a heme iron acquisition gene and involves in heme utilization. The strains that carry *chuA* are able to survive and persist inside macrophages, which can be suggested as a major contributor to the multiplication of *E. coli* strains in the inflamed human intestines. Importantly, the upregulating of the *chuA* stimulates the release of TNF-α, which may promote dysbiosis and microbe-driven intestinal inflammation (50). *E. coli* strains related to phylogroups B2 and D carry more virulence-associated genes compared to *E. coli* related to the other phylogroups. Furthermore, B2 and D phylogroups usually possess certain virulence factors, which can lead to extraintestinal infections and also give them the ability to persist within the human gastrointestinal tract (51). Dadi et al. reported that *E. coli* strains which are engaged in extraintestinal infections, including urinary tract infections (UTIs) are most likely to belong to phylogroup B2 or, to a lesser degree, to phylogroup D (52). Moreover, IBD patients with anorectal complications, which include perianal abscesses and anal fistulas, appear to have an increased risk for UTIs. It could be explained that in these patients anorectal complications could lead to bacterial translocation from the perineum to the bladder (53, 54). Accordingly, the overgrowth of *E. coli* strains with extraintestinal virulence capacity in IBD patients could act as a risk factor for UTI development. Martinez-Medina et al. (55) noticed that IBD-associated *E. coli* strains, which have biofilm-producing capacity, mainly belong to B2 phylogroup. Clinical observations revealed that the density of the mucosal biofilm on intestinal tissues was higher in IBD patients than in patients with irritable bowel syndrome (IBS) or controls (56, 57). Biofilm formation by *E. coli* strains especially AIEC in human gut gives the strains an advantage for intestinal colonization and consequently increases their chance to invade the intestinal epithelium and further results in mucosal inflammation (55). Although several studies have reported increased colonization by strains belonging to phylogroups B2 and D in IBD patients (58–60), some authors indicated a similar distribution of phylogroups among IBD and healthy cohorts (6, 61).

In the current study, PCR pathotyping analysis revealed that ETEC and DAEC were the prevalent pathotypes, where *lt* gene was detected in 21 (15.9%) *E. coli* isolates, and *daaD* was identified in 11 (8.3%) isolates. An important insight of our study is a relatively higher prevalence of ETEC pathotype than that reported by others; in which Meheissen et al. reported the detection of *lt* in two out of 60 (3.3%) and Kmetova et al found five *lt* out of 437 (1.2%) samples tested. Previously, Brubaker et al. described that ETEC infection significantly induces intestinal and systemic inflammation among subjects who remained asymptomatic (62). Notably, an important insight of the mentioned study was the extent to which asymptomatic ETEC infection could cause significant intestinal inflammation that may lead to further inflammation-mediated GI disorders such as IBD. More according to a large-scale study by Eybpoosh et al. ETEC was the second most frequent *E.coli* pathotype in Iran and was detected in all investigated provinces including twenty-nine cities (63). We suggest that the high prevalence of ETEC among IBD patients in our study could support the potential role of ETEC strains in inducing intestinal inflammation and their impact on IBD progression in such endemic regions.

Though still controversial, some preliminary evidence suggests the link between DAEC and UC. In, Burke and Axon reported that a majority of isolated *E. coli* strains from the stool of UC patients were DAEC pathotypes with both enteropathogenic and enterotoxigenic properties, on the contrary to the isolates from healthy subjects (64). DAEC strains harboring Afa/Dr have been indicated to adhere to the colonic epithelium of UC patients and to induce proinflammatory cytokines TNF-α and IL-8 *via* the interaction of their fimbriae with membrane-bound host receptors (65). Additionally, some research on the pathogenesis of DAEC indicates that Afa/Dr fully differentiated epithelial cells, inducing the rearrangement of brush border-associated cytoskeletal proteins F-actin followed by the loss of the epithelial cell microvilli (66). In the present work, EIEC and STEC were not detected in the collected samples from CD and UC patients. Importantly, however, there is no epidemiological evidence directly linking the colonization of DAEC to the development and progression of UC. Accordingly, more large-scale case-control studies are needed to clarify the potential role of *E. coli* pathotypes in the pathogenesis of IBD.

In the present study, we found that approximately half of the cultured *E. coli* strains from UC and CD patients were non-susceptible to three or more antimicrobial categories and would be categorized as MDR. To our knowledge, only a very small fraction of studies have reported the antimicrobial resistance patterns of Enterobacteriaceae strains among IBD patients. In a study conducted by Martinez-Medina et al., it was reported that mucosa-associated *E. coli* isolates from CD patients were more frequently resistant to β-lactams than those isolated from the intestine of control subjects (67). Dogan et al. reported that CD-associated *E. coli* frequently manifests resistance to commonly used antimicrobials, particularly, resistance to ciprofloxacin, rifaximin/rifampin, and trimethoprim/sulfamethoxazole (68). They also found that most of the *E. coli* isolates from CD patients were MDR compared to those from healthy subjects.

More importantly, 69% of *E. coli*-colonized patients with IBD harbored strains that were resistant to ciprofloxacin, which is commonly used as empirical antibiotic therapy to treat IBD flare. This rate of ciprofloxacin-resistant *E. coli* among Iranian IBD patients is significantly higher than those found in the study performed by Dogan et al., in which 29% of *E. coli*-colonized ileal CD patients harbored *E. coli* with resistance to ciprofloxacin (68). Interestingly, we found that ciprofloxacin-resistant *E. coli* were more common in the IBD patients with prior use of ciprofloxacin as antibiotic therapy for IBD flare (73.3%) compared to those patients without adjunct ciprofloxacin therapy (47.5%). Ciprofloxacin is used in combination with metronidazole in the treatment of IBD patients, especially in severe cases or during flare-ups (69). Some previous studies have suggested ciprofloxacin is an adjunct to infliximab (73% vs. 39%, *P* = 0.12) or adalimumab (71% vs. 47%, *P* = 0.047), and had a higher response than these two biologics alone (70, 71). In addition to recent studies demonstrating the association of AIEC pathotype with CD, and the ability of ciprofloxacin to eradicate bacteria within macrophages, several investigators suggest that ciprofloxacin may be particularly useful for treating IBD (69). Moreover, patients with ileal or ileal with right-sided CD and circulating antibodies directed against *E. coli* have been shown to have a higher response rate to budesonide plus ciprofloxacin than the group with no antibody predominant profiles (72). However, our findings demonstrate substantial implications for this strategy. Hence, we suggest that antimicrobial resistance along with non-targeted antimicrobial selection could contribute to IBD flare-ups related to overgrowth of ciprofloxacin-resistant *E. coli* in those who have received ciprofloxacin for the long term.

## Conclusion

In the present study, we found that *E. coli* strains colonize the gut of Iranian patients with IBD most frequently belonged to phylogenetic groups B2 and D. We also conclude that *E.coli* isolates from IBD patients have been revealed to be resistant to commonly used antibiotics, in which most of them harbored strains that would be categorized as MDR. We also consider the possibility that the colonization with ciprofloxacin-resistant *E. coli* strains may arise due to the empirical therapy. We suggest that antimicrobial therapy in IBD patients should be informed by knowledge of antimicrobial susceptibility of the gut resident Enterobacteriaceae. However, there are some limitations in this study. First, a relatively low sample size of CD patients and the single center recruitment, could be technical impediments that can adversely influence the statistical power of the results. In addition, the lack of healthy control group and the lack of follow-up of patients are other limitations of the present work. Further research should be conducted to investigate the characteristics of mucosa-associated *E. coli* especially AIEC pathotype and their contribution to the inflammatory process in Iranian patients with IBD.

## Data availability statement

The original contributions presented in this study are included in the article/Supplementary material, further inquiries can be directed to the corresponding authors.

## Ethics statement

The studies involving human participants were reviewed and approved by the Institutional Ethical Review Committee of Research Institute for Gastroenterology and Liver Diseases (RIGLD) at Shahid Beheshti University of Medical Sciences (Project No. IR.SBMU.RIGLD.REC.1399.052). The patients/participants provided their written informed consent to participate in this study.

## Author contributions

BFN and BN performed the sample collection and microbiological experiments. BFN and HH wrote the manuscript draft. AY contributed to the study design, conceptualization, and methodology. SS and MA contributed to the colonoscopy procedures and provided clinical consultations. AY and GE provided the important intellectual content and critically revised the manuscript. All authors read and approved the final version of the manuscript and authorship.

## Abbreviations

AIEC: adherent-invasive *E. coli*
BHI: brain heart infusion
CD: Crohn’s disease
CDAI: Crohn’s disease activity index
DEC: Diarrheagenic *E. coli*
DAEC: diffusely adherent *E. coli*
EAEC: enteroaggregative *E. coli*
EIEC: enteroinvasive *E. coli*
EPEC: enteropathogenic *E. coli*
ERIC-PCR: enterobacterial repetitive intergenic consensus sequence polymerase chain reaction
ETEC: enterotoxigenic *E. coli*
ExPEC: extraintestinal pathogenic *E. coli*
IBD: inflammatory bowel disease
MDR: multidrug-resistant
MLST: multilocus sequence typing
PBS: phosphate buffer solution
PFGE: pulsed-field gel electrophoresis
RAPD: randomly amplified polymorphic DNA
STEC: Shiga toxin-producing *E. coli*
UC: ulcerative colitis
UTIs: urinary tract infections.
