# Supplementary material for: Phylogrouping and characterization of Escherichia coli isolated from colonic biopsies and fecal samples of patients with flare of inflammatory bowel disease in Iran
Source: Front Med (Lausanne). 2022 Aug 29;9:985300. doi: 10.3389/fmed.2022.985300 (PMC9464868; doi:10.3389/fmed.2022.985300)
Supplement: Supplementary file 1 [file Table_1.DOCX]

**Table S1** Distribution of each phylogroup of *E. coli* isolates within IBD patients.

| **Phylogenetic groups** | **UC**  **(*n* = 112)** | **CD**  **(*n* = 20)** |
| --- | --- | --- |
| A  B1  B2  C  D  E  F | 17  15  25  10  25  10  10 | 3  2  7  0  5  1  2 |

*IBD* inflammatory bowel disease, *UC* Ulcerative colitis, *CD* Crohn’s disease.
